# Dealing with the Effect of Air in Fluid Structure Interaction by Coupled SPH-FEM Methods

**DOI:** 10.3390/ma12071162

**Published:** 2019-04-10

**Authors:** Cristiano Fragassa, Marko Topalovic, Ana Pavlovic, Snezana Vulovic

**Affiliations:** 1 Department of Industrial Engineering, University of Bologna, Viale Risorgimento 2, 40136 Bologna, Italy; cristiano.fragassa@unibo.it; 2 Faculty of Engineering, University of Kragujevac, Sestre Janjic 6, 34 000 Kragujevac, Serbia; topalovic@kg.ac.rs (M.T.); vsneza@kg.ac.rs (S.V.)

**Keywords:** fluid–structure interaction, SPH, FEM, water impact, air effect, drop test

## Abstract

Smoothed particle hydrodynamics (SPH) and the finite element method (FEM) are often combined with the scope to model the interaction between structures and the surrounding fluids (FSI). There is the case, for instance, of aircrafts crashing on water or speedboats slamming into waves. Due to the high computational complexity, the influence of air is often neglected, limiting the analysis to the interaction between structure and water. On the contrary, this work aims to specifically investigate the effect of air when merged inside the fluid–structure interaction (FSI) computational models. Measures from experiments were used as a basis to validate estimations comparing results from models that include or exclude the presence of air. Outcomes generally showed a great correlation between simulation and experiments, with marginal differences in terms of accelerations, especially during the first phase of impact and considering the presence of air in the model.

## 1. Introduction

The finite element method (FEM) represents a powerful and flexible numerical procedure, able to provide a response to a large range of engineering problems [1], including the design of aeronautics and maritime vessels [2,3] or other similar applications where an attention to the fluid–structure interaction (FSI) is fundamental. In [4], for instance, the use of finite elements (FEs) was reported in investigating the multi-physics in the case of ship’s seaway response against wave loadings, including fore and aft slamming. In [5,6], the FEM permits the parametric design of an aircraft wing, using the FSI as an approach for improving efficiency and accuracy in the analysis.

In brief, when dealing with FSI, FEM represents an extremely efficient tool for investigation; at the same time, whenever geometrical nonlinearity significantly affects the structural behavior, the co-rotational FE formulations [7,8] may still keep the computations highly efficient [9]. When a FSI emerges as predominant in an analysis, the utilization of tools based on finite elements (FE) is often followed by certain difficulties in the calculation, primarily linked to the presence of large deformations in the fluid surrounding the structure [10].

During the design of the aircraft fuselage, for instance, the engineers should be concerned in foreseeing its behavior in extreme situations, such as a crash landing on the water surface [11]. But, to accurately predict this aircraft crashworthiness [12], FEM has to be coupled with a mesh-free method (MFM) as in [13] with the scope to overcome the FE limitations.

Following the example of the aircraft during a crash landing on a hard surface (e.g., ground), the load is directly transferred to the fuselage support structure with effects that can be investigated by FE [12,13]. Crashing on a water surface also creates a pressure gradient applied to the fuselage outer skin that can easily lead to its rupture [14], causing a fast sinking [12], but these phenomena cannot be investigated without coupling FEM with other methods (as in [6]). This methodological necessity emerged during the years after several different attempts.

In general, the state of bodies entering in liquids represents a wide-ranging problem that started to attract the interest of naval architects and designers since 1920s with the works of Wagner [15] and Von Karman [16]. Both researches considered the simplified case of a 2D rigid body and purely hydrodynamic effects, but these provisional results already demonstrated their relevance in procedures, such as ship design. With [17], the local hydroelastic effects were also considered, where the theory was used to investigate the local stresses induced by slamming on a vessel. The structure under examination was simplified adopting a 2D Timoshenko beam as a model while the Wagner theory helped to represent the surrounding fluid. In particular, water was presumed as incompressible and several boundary effects, such as air entrapment and cavitation, were neglected. Despite these assumptions, the proposed theory was quite complex and a further simplification was needed to allow a larger applicability [18]. Following that stage, experimental measures substantially confirmed the validity of the theory [19]. As a consequence, a new class of methods and tools was available for the researchers working towards a better comprehension of the FSI phenomena.

Focusing the attention on the emerging numerical methods in this field of investigation, one of the first uses was proposed by [17], and followed by [18], where the situation of a wedge impacting on the water surface was analyzed solving non-linear equations by numerical solvers. Nevertheless, [19] demonstrated that it is possible to merge a method based on FE and validate over the structural domain with Wagner’s theory, applicable on the fluid domain. At that point, as the final step, it was necessary to develop a numerical approach able to model this fluid domain and the related equations. 

Several solutions were proposed in the following years to fill the methodological gap. In particular, in [20,21,22], the boundary element method (BEM) was proposed referring to the fluid coupled with FEM for the structure. This integration was validated by experimental data in [23]. 

In a wide-ranging term, the role of hydroelasticity in water entry problems was discussed in detail in [24], applying alternative methods. The work continued to take into account a two-dimensional geometry, but offered some valid considerations, both in the cases of rigid and elastic bodies. Evidences of numerical estimations were provided using self-developed and commercial codes. In particular, MSC Dytran and LS-DYNA software packages were specifically recommended since their algorithms deal with the coupled hydroelastic interaction with the possibility to model the effect of air cushioning. Furthermore, in [24] it was also highlighted how the results proposed by these two numerical tools are almost equivalent, since they use the same equations for describing the states of water and air. The aforementioned literature-based broad confidence in LS-DYNA predictions, supported the decision to prefer its FSI modeler for the present research.

According to [25,26], LS-DYNA algorithms permit a FE modeling procedure that correctly takes into account multi-material formulations, penalty contacts, and hydrodynamic loads. However, no suggestion was provided referring to the specific MFM to be integrated. Among the more than 30 different mesh-free methods [27], this research favored an approach that couples FEM with smoothed particle hydrodynamics (SPH). The SPH, in fact, seems particularly suitable for investigating the effect of water impact on structures (when coupled with FEM) as described in [28]. 

The SPH was originally created for analyzing astrophysical problems [29,30] and later extended to solve computational fluid dynamics (CFD) problems, governed by the Navier–Stokes equations [31] and computational solid mechanics (CSM) [32,33]. The SPH approach is based on the continuum mechanics and the Lagrangian material framework, meaning that the motion of the particular fluid section is observed [34,35]. A significant overview of SPH methods, together with its recent developments and applications in numerical algorithms are given in [36].

Whereas SPH-FEM coupling is often seen in relevant scientific literature [37,38], different commercial software codes also implement this integrated method. For instance, ABAQUS uses SPH functions to overcome the mesh distortion problem [39], while a wider range of applications can be found as implemented in LS-DYNA [40]. 

At the same time, due to the high computational requirements inherent of the SPH method [40], when researchers and engineers deal with complex situations such as aircraft ditching, the influence of air is commonly neglected and problems are investigated only considering the interaction between structure and water. For instance, in [41], the elastic deformation of a marine propeller was analyzed using the normal modes method, and the water flow field was considered using the matched asymptotic expansions method. This and similar studies did not account for the air–water interaction or for viscous effects. As also shown in the present work, this simplification does not affect the accuracy of stress prediction, since the presence of air in the prediction model does not influence the contact loads between water and object, but introduces other limits instead.

Therefore, at least during the initial phases of fuselage design, the precaution of modeling the interaction with air does not represent a relevant factor. On the other hand, in later stages of the design process (e.g., safety assessment), this inclusion could be necessary for a real evaluation of fuselage crashworthiness after the initial impact [42]. At this point, in fact, the integration between SPH and FEM could come in really useful: Thanks to this combination between the two approaches, it is possible to correctly predict effects such as air bubbles sticking to the exterior surface [43,44], a phenomenon able to increase the overall buoyancy. 

In addition, since the presence of air particles can represent a physical obstacle in respect to the water penetration, the inclusion of air particles in the model is a necessary step for a better prediction of the real evolution of aircraft ditching [44,45,46]. 

Finally, an integration between SPH and FEM was also demonstrated as a valid solution to predict the cavitation in low pressure areas, which is manifested as a function of the gap between SPH particles [40,42,47,48]. 

## 2. Materials and Methods

This paper proposes a numerical model for performing water impact simulations in the case of a fluid–structure interaction (FSI), able to take into consideration the presence of two fluids, namely air and water. It is based on a combination of SPH and FEM techniques, implemented by the commercial solver LS-DYNA R9.0.1. Pre- and post-processing was done in LS-PrePost 4.3. In particular, the effects of the presence of air are investigated in terms of FSI, together with the numerical replicability of those effects. Numerical models alternatively exclude and include the air, while results are related to each other in the way to examine the physical phenomena they are able to detect. Numerical estimations are also compared with experiments and, specifically, with data available in [42] and [43]. These measures, acquired by drop tests performed in our laboratory in the past and detailed in [42], are now reconsidered and used to direct the simulation, defining the overall conditions and validating the research.

### 2.1. Geometry

The experiment consisted of a series of free falls of a flexible cylinder in water. Impacts were performed varying the drop height from 250 to 1500 mm. Special equipment was developed for the scope. Two vertical rails of 3000 mm were fixed to the roof of the laboratory and to a water tank. This tank, 2000 mm large, 1750 mm long, and 1000 m deep (filled up to 600 mm), was dimensioned in this way to minimize the border effects. An aluminum sledge, running over the rails, held the cylinders during the free fall in a perfect verticality; Teflon inserts prevented the cylinder from tilting during the fall by getting in contact with the rails. A total inertial mass of approximately 3.1 kg, made by the cylinders together with all the accompanying parts and material, was considered.

**Figure 1:**
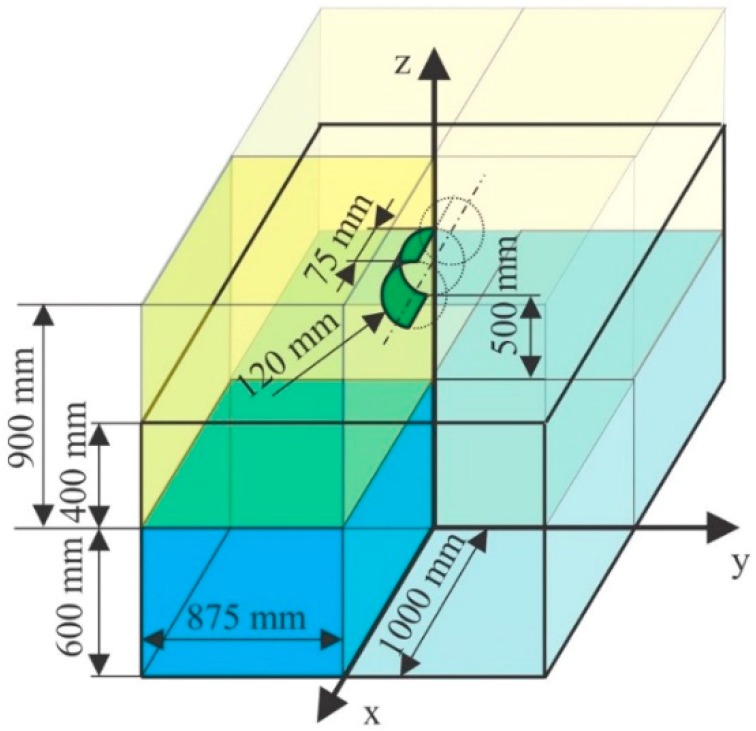
Geometrical configuration, dimensions, and symmetries.

The present paper reproduces the experiment in a numerical way. Figure 1 exhibits the system under investigation, together with all its geometrical specifications. In particular, in the figure it is possible to see the case of the cylinder located at 500 mm over the water.

Due to symmetry, only a quarter of the model was numerically processed, applying mirror boundary conditions placed on external SPH surfaces, and FEM nodes on mirror planes with constrained displacement in *x* or *y* directions. Mirror planes are defined in LS-DYNA using the *BOUNDARY_SPH_SYMMETRY_PLANE keyword with the effect of generating a ‘ghost particle’ for each SPH particle close to the plane. These ghost particles are used to maintain the kernel compactness as also mentioned in [49], while due to the nature of the SPH method, the free surface boundary condition is naturally satisfied [49]. The cylinder FEM modes that lay on the symmerty planes are constrained using the *BOUNDARY_SPC_SET keyword.

### 2.2. Materials Properties

The cylinder impacting on water was made of three layers of plain weave fabric E-glass with a nominal weight of 480 ± 20 g/m^2^. The matrix is epoxy and the fiber volume fraction was 60%. According to the material data from [42], a nominal flexural modulus (*E*) of 33 GPa, a Poisson’s ratio (*ν*) of 0.28, and a density (*ρ*) of 2540 kg/m^3^ were adopted.

Regarding the water and the air, a density of, respectively, 1000 kg/m^3^ and 1.22 kg/m^3^, was considered, while the viscosity coefficient was defined as 10^−6^ m^2^s^−1^. Other values for these fluids (such as pressure cutoff, relative volume, Young’s modulus, and Poisson’s ratio) kept a default value of zero.

The mechanical properties of the cylinder, water, and air were defined in LS-DYNA using, respectively, the *MAT_ELASTIC_TITLE; *MAT_NULL_TITLE and *MAT_NULL_TITLE models. 

### 2.3. Kernel Approximation

Two alternative situations were modeled, without air and with air; in this second case, a layer of 900 mm of air above the level of water and around the cylinder was added. 

SPH was used to model both fluids (water and air) and to take into account their interaction with the surfaces of structures, discretized by shell finite elements [39], but also their mutual interaction. SPH uses discretization of the continuum into pseudo-particles somewhat similar to finite elements, but without common nodes among them [34,35]. These pseudo-particles can have different neighbors throughout the analysis, which enables better handling of large deformations. The downside is that mesh-free lagrangian methods require frequent searches for neighboring particles. To solve conservation laws of continuum mechanics in SPH, two principles are used: kernel and particle approximations [36].

The conservation laws of continuum mechanics are defined by partial differential equations, which in SPH are transformed into integral equations by an interpolation function that provides the “kernel estimate” of the field variables at a specific point [13]. 

For any SPH pseudo-particle with position vector x, a function f(x) defined within the domain Ω can be expressed in an integral form as:

(1) $$f\left(x\right)=\int_{\Omega }^{}f\left(x^{\prime }\right)\delta \left(x-x^{\prime }\right)dx^{\prime },$$

where $x^{\prime }$ is the position vector of the material point in a domain Ω and $\delta \left(x-x^{\prime }\right)=\{\begin{array}{cc} 1 & x=x^{\prime }\\ 0 & x\neq x^{\prime } \end{array}$ is the Dirac delta measure [19].

Replacing the Dirac delta measure with a bell-shaped kernel function $W\left(\left|x-x^{\prime }\right|,h\right)$ where h is the smoothing length, a kernel approximation of the function f(x) is given by:

(2) $$\langle f\left(x\right)\rangle =\int_{\Omega }^{}f\left(x^{\prime }\right)W\left(\left|x-x^{\prime }\right|,h\right)dx^{\prime }.$$

### 2.4. Particle Approximation

Since continuum mechanics infer the division of the matter in a finite number of pseudo-particles that carry specific mass and occupy a specific space, continuous integral equations given with Equation (2) can be transformed into discrete forms by the summation over all pseudo-particles within the support domain defined by the smoothing length *h* [34]. Infinitesimal volume $dx^{\prime }$ from Equation (2) is replaced by finite volume of the particle *j*, which can be calculated using its mass and density through $\Delta V^{j}=m^{j}/\rho^{j}$, hence, the discretized particle approximation of a function f(x) for particle *i* is:

(3) $$\begin{array}{l} \langle f\left(x_{i}\right)\rangle ≅\sum_{j=1}^{NNP}f\left(x^{j}\right)W\left(\left|x^{i}-x^{j}\right|,h\right)\Delta V^{j}\\ =\sum_{j=1}^{NNP}\frac{m^{j}}{\rho^{j}}f\left(x^{j}\right)W\left(\left|x^{i}-x^{j}\right|,h\right) \end{array}$$

where *NNP* is the number of nearest neighboring particles, while $x^{i}$ and $x^{j}$ are position vectors of observed particle *i* and neighboring particles *j*, respectively.

### 2.5. SPH for Viscous Fluids

Since in the previous section superscripts *i* and *j* are used to denote particles, in this section the Greek subscripts α,β,γ are used to denote coordinate directions. The total stress tensor $\sigma_{\alpha \beta }$ in a viscous fluid consists of hydrostatic pressure p and viscous shear stress $\tau_{\alpha \beta }$ and is represented by Equation (4):

(4) $$\sigma_{\alpha \beta }=-p\delta_{\alpha \beta }+\tau_{\alpha \beta },$$

where $\delta_{\alpha \beta }$ represents the Kronecker delta symbol [34].

If the components of the fluid velocity are denoted with $v_{\alpha }$, considering Einstein’s summation convention, viscous shear stress $\tau_{\alpha \beta }$ can be calculated by Equation (5):

(5) $$\tau_{\alpha \beta }=\mu \left(\frac{\partial v_{\beta }}{\partial x_{\alpha }}+\frac{\partial v_{\alpha }}{\partial x_{\beta }}-\frac{2}{3}\delta_{\alpha \beta }\frac{\partial v_{\gamma }}{\partial x_{\gamma }}\right),$$

where μ represents the dynamic viscosity coefficient [50]. Using kernel and particle approximation on Equation (5), with some rearranging [34], viscous shear stress in SPH can be calculated using:

(6) $$\tau_{\alpha \beta }=\mu \left[\begin{array}{l} \sum_{j=1}^{NNP}\frac{m^{j}}{\rho^{j}}v_{\beta }^{ji}\frac{\partial W^{ij}}{\partial x_{\alpha }^{i}}+\sum_{j=1}^{NNP}\frac{m^{j}}{\rho^{j}}v_{\alpha }^{ji}\frac{\partial W^{ij}}{\partial x_{\beta }^{i}}\\ -\left(\frac{2}{3}\sum_{j=1}^{NNP}\frac{m^{j}}{\rho^{j}}v^{ji}\cdot \nabla_{i}W^{ij}\right)\delta_{\alpha \beta } \end{array}\right],$$

where $W^{ij}$ is a short notation of the kernel function $W\left(\left|x^{i}-x^{j}\right|,h\right)$. Equation (6) is a general form and can be applied to compressible fluids such as the air. For incompressible fluids (e.g., water), Equation (6) can be simplified to the following form:

(7) $$\tau_{\alpha \beta }=\mu \left[\sum_{j=1}^{NNP}\frac{m^{j}}{\rho^{j}}v_{\beta }^{ji}\frac{\partial W^{ij}}{\partial x_{\alpha }^{i}}+\sum_{j=1}^{NNP}\frac{m^{j}}{\rho^{j}}v_{\alpha }^{ji}\frac{\partial W^{ij}}{\partial x_{\beta }^{i}}\right],$$

A high turbulence effect can be found in several situations, such as a wave breaking on the coast or the prediction of tsunami hazard mitigation along the coastal regions. In these cases, the inclusion of corrections in the standard SPH, with the aim at taking care of the turbulence in the numerical model, represents an essential step for solving the problem [51]. In the situation of interest, represented by a light weight cylinder, impacting on water with a low entry velocity, the turbulence effect was considered as secondary. Then, a standard SPH formulation, with a proper dimension of SPH particles, was preferred to describe the overall behavior of water and air, including the turbulent movements of their particles.

### 2.6. Contacts in a Coupled FEM-SPH 

**Figure 2:**
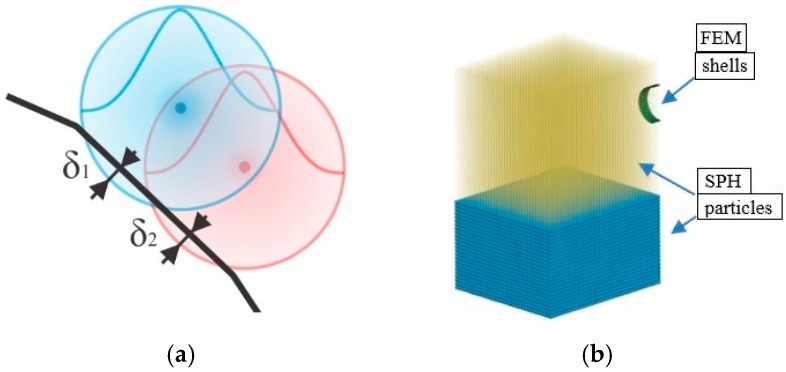
Modeling the contacts: (**a**) shell–particle and particle–particle contacts (δ1 and δ2 as penetration depths); (**b**) finite element method (FEM) shell mesh immerged (the cylinder) in a Smoothed particle hydrodynamics (SPH) environment (air and water).

The correct contact definition in explicit-code simulation software represents an essential aspect to assure the accurate determination of load transmission, and subsequently, stress calculation [50,52,53]. In the present case, both shell–particle and particle–particle contacts were considered (Figure 2). Based on penetration depth (e.g., δ1 and δ2 in Figure 2a), contact forces between shell elements and SPH particles can be calculated.

The interaction between the FE structure (the cylinder) and the SPH particles (representing air or water) was implemented in LS-DYNA by *CONTACT_AUTOMATIC_NODES_TO_SURFACE contact type (as suggested in [39]), defining the master–slave penalty algorithm in which the FEM shell surface was a master and SPH particles slaves. All parameters for the contact type definition were set to the default values (Table 1) in accordance with similar analyses, except for the scale factors slave (SFS) and the scale factors master (SFM). In particular, for a better representation of the contact characteristics, these factors were defined as SFS = 1.0 and SFM = 1.0 in the case of contact between cylinder and water, and SFS = 0.5 and SFM = 0.5 between cylinder and air [14,39].

Due to the nature of SPH method (i.e., smoothing of variable values within a bell-shaped particle domain), the contact between two fluids with a density ratio larger than 10, as in this case, cannot be easily handled by the standard SPH interpolation [40]. A two-phase SPH model with artificial cohesion and background pressure was proposed in [54,55,56], but such an approach leads to a significant increase in the computational complexity and time. Furthermore, effects of negative pressure or pressure noises can emerge if the background field of pressure is not correctly chosen [54,55,56]. The present approach, on the other hand, considers air and water as two different models. Penalty contact forces are used to model the interaction between air and water particles. Consequently, when the interpolation of values is performed inside the SPH algorithms, only particles from the same material (air or water) are initially considered. Afterwards, when a particle under consideration is found in contact with a particle from a different material, an additional contact force is calculated and added. In this way, the interaction between diverse materials is modeled. Therefore, the LS-DYNA embedded node-to-node penalty contact algorithm DEFINE_SPH_TO_SPH_COUPLING was considered [41] and default parameters were preferred when possible (Table 2). The penalty scale factor for contact damping coefficient was set to 0.7 (as in [14,25,39]) where the master part was represented by water particles and slave part by air particles in this new situation.

### 2.7. System Discretization

The 3D-domain discretization (Figure 2b) consisted of:

- 76 shell elements, 1.8 mm thick, with a total amount of 100 nodes, for the flexible cylinder;
- 33,600 particles for the water;
- 50,500 articles for air, when considered.

FEM properties for shell elements were defined inside the *SECTION_SHELL_TITLE, where all values remained as set by default and thickness was set to millimeters. SPH properties were defined in *SECTION_SPH_TITLE. In particular, the smoothing length for both water and air was set to 1.0, 1.1, and 1.2 in order to test the influence of smoothing length on computation time and fluid behavior. This was done against the LS-DYNA recommendations that suggest limiting this value between 1.05 and 1.30. This exceptional choice was made to prevent an averaging of state variables, to enable tearing, and ease the mixing of water and air fluids. Finally, scale factor for the minimum and maximum smoothing length was also set to 1.0 in the case without air or 1.2 with air [33,37], values which prevented LS-DYNA from changing the smoothing length. 

The effect of gravity along the Z-axis was introduced using the *LOAD_BODY_Z. The velocity of impact was specified by *BOUNDARY_PRESCRIBED_MOTION_SET and load curves. By changing the values in *DEFINE_CURVE_TITLE, different impact conditions were investigated.

## 3. Numerical Results

Four simulations were implemented and presented here in detail; they differ for the drop heights, 500 mm and 1500 mm and, per each height, repeated with and without the air effect. Numerical predictions were compared with experimental ones, coming from a second elaboration of measures from [42] in terms of accelerations and deformations. 

The drop-test can be ideally split into two phases, distinguished by the moment of impact: the free fall through the air and the penetration into the water.

### 3.1. Free Fall

**Figure 3:**
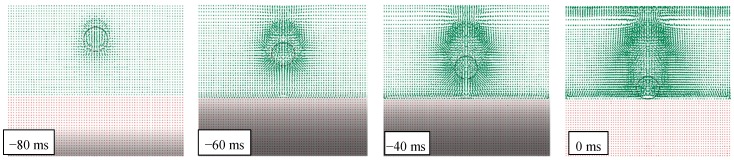
FEM-SPH model used for simulating the air effect during the free fall (drop height of 1500 mm).

The free fall is a special kind of motion in which the only force acting upon an object is the gravity, with Earth’s gravitational acceleration equal to 9.81 m/s^2^. At the same time, as an object falls through the air, it meets a resistance related to the presence of air. This resistance is related to the collisions between the object and the air molecules, together with their inertia in being crossed. The effect of air resistance is dependent upon several factors that often have a negligible effect on the impact, and it is frequently ignored or merely considered for correcting the speed/energy of impacting objects by empirical formula. For instance, in Figure 3 it is possible to see the track that creates in the air the passage of the object during the free fall, leaving a general disorder in the molecules. Furthermore, it is possible to note how the pressure increases in front of the object, as it progressively approaches the impact surface, visible as an increase in the concentration of the particles. 

Furthermore, the effect on the vertical column of air, is visible as rarefaction of the particles and their general movement downward to follow the falling object. A rebound effect is also evident with particles of air moving in the way to close off the space left empty by the descending object after its passage, even with ascent movements of molecules. 

### 3.2. Water Penetration

**Figure 4:**
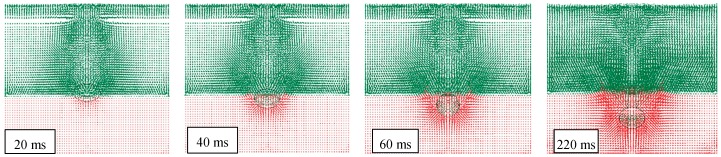
FEM-SPH model used for simulating the water penetration (drop height of 1500 mm).

At the conventional time *t_0_* = 0 ms, the impact occurs and the water penetration starts. Figure 4 highlights this sequel in the case of a drop height of 1500 mm and the presence of air. 

It can be observed that after 20 ms, a portion of the object, approximately one third of the cylinder volume, has been already entered in water, reducing object speed and energy. Also, the water particles are almost undisturbed in their original position, except for a very limited layer, closer to the object surface. This means that the impact has been mainly absorbed by the object through deformation. 

After an additional time of 20 ms, another one third of the object is penetrated in water, and the particles have been significantly influenced by the pressure wave and this effect grows up in time. Since 60 ms, the whole object is submerged and starts its deceleration into the deep water. At the same time, waves and ripples appear on the water’s surface, together with a condition of mix and interaction between particles of two fluids.

## 4. Simulation vs. Experiment

**Figure 5:**
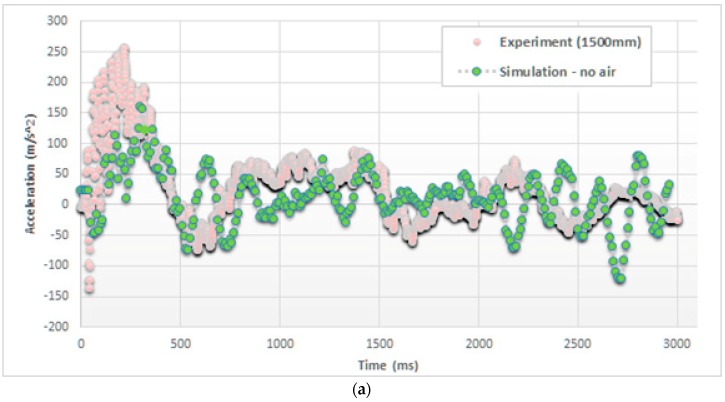

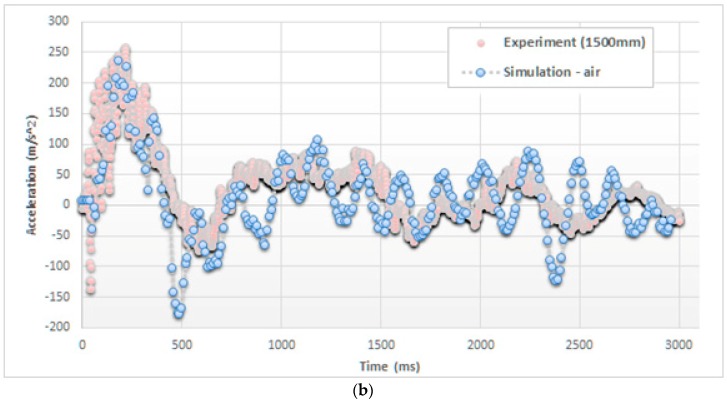
Comparison of acceleration on the top of the cylinder (drop height of 1500 mm), (**a**) without and (**b**) with considering the air effect.

The model validation was performed comparing the accelerations, measured and simulated during the impact, exactly at the top of the cylinder, in the case of drop heights of 500 mm and 1500 mm. In particular, Figure 5 reports the trends of these accelerations considering both models, without and with the air effect, in the case of 1500 mm drop height. 

### 4.1. Accelerations

A dual effect has to be considered: (i) The deceleration of the object as it comes into contact with water and (ii) the deformation of the elastic cylinder. As a result, a superposition of harmonic oscillations is expected [34], with fast changes in the acceleration values.

Accordingly, from experiments it can be observed that the peak of acceleration, approx. 250 m/s^2^, is reached in approx. 200 ms after the impact (namely at *t_0_* = 0 ms). Then, this acceleration decreases toward a negative peak, approx. −175 m/s^2^, and an additional 300 ms, before starting to oscillate within a damping effect. 

Comparing the trends, as it can be seen in Figure 5, the inclusion of air particles in the model significantly changes the impact behavior in terms of accelerations. In particular, SPH air particles amplify pressure peaks, providing more realistic maximum values of accelerations (e.g., 240 vs. 160 m/s^2^). 

**Figure 6:**
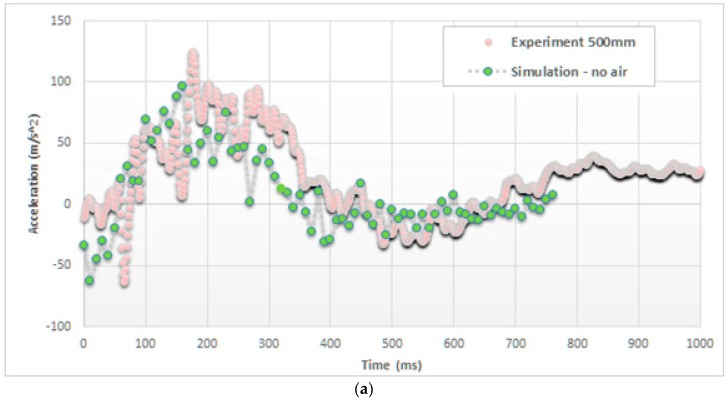

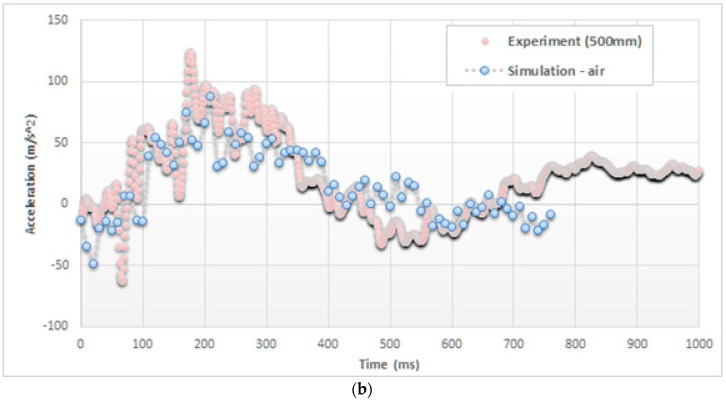
Comparison of acceleration on the top of the cylinder (drop height of 500 mm), (**a**) without and (**b**) with considering the air effect.

Figure 6 reports the same trends of accelerations in the case of a drop height of 500 mm and focuses the attention on the initial part of the impact (up to 750 ms).

### 4.2. Deformations 

The following figures offer several details on the phenomena emerging during the first moments of impact. It is the case of a cylinder falling down from a drop height of 1500 mm and from images that some visual results can be estimated in terms of cylinder deformations. 

**Figure 7:**
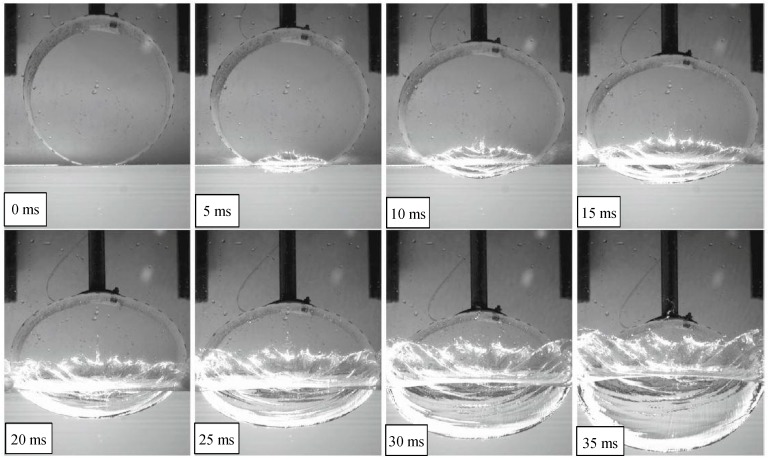
High speed images of a cylinder falling from a drop height of 1500 mm after the impact [42].

**Figure 8:**
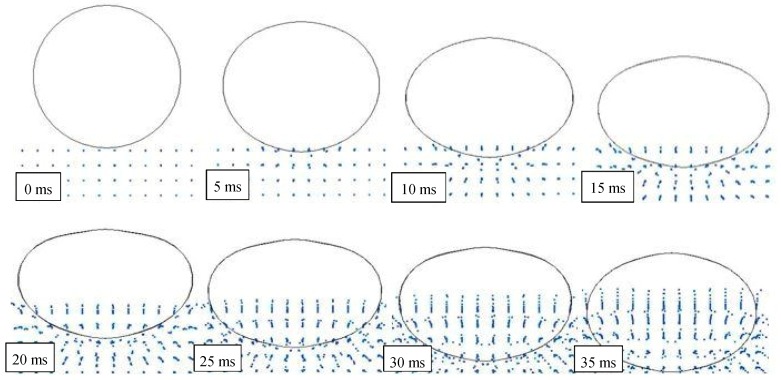
Impact numerical results without considering the air effect (drop height of 1500 mm).

**Figure 9:**
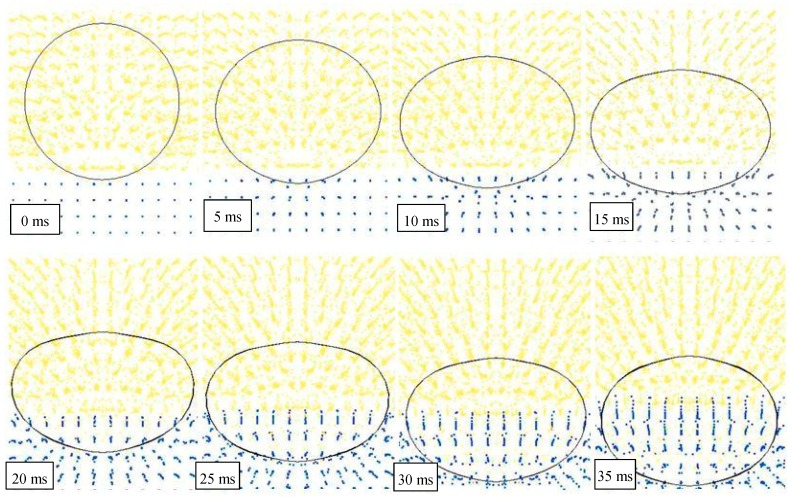
Impact numerical results considering the air effect (drop height of 1500 mm).

In particular, Figure 7 shows high speed images of the cylinder during the impact [41], while Figure 8 and Figure 9 report the profile of the objects in the same physical conditions, as obtained by numerical simulation, respectively, without and with considering the air effect.

**Figure 10:**
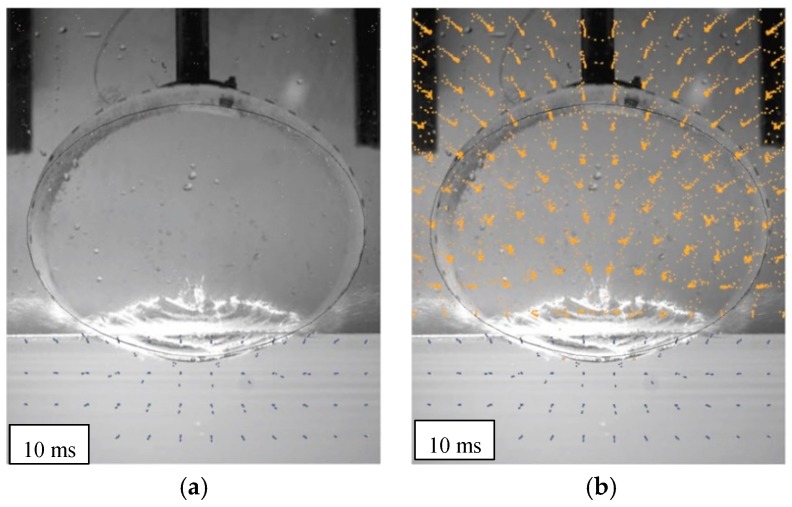
Comparing the effect of air on prediction (drop height of 1500 mm): (**a**) without air; (**b**) with air.

Overlapping the experimental and numerical results, as shown in Figure 10, in the case of 10 ms, it is possible to better understand the correspondence between the results. Discrepancies in the shape of the top part of the object, are mainly caused by the presence of a rigid fixture, not modeled by FE, that alters the free deformation of the cylinder during the impact. 

**Figure 11:**
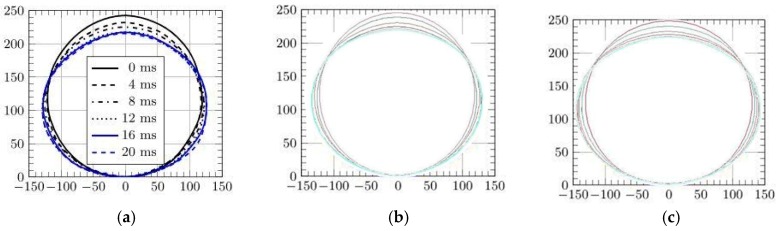
Deformation of the cylinder falling from 500 mm [42]; (**a**) experiments, (**b**) without air, (**c**) with air.

**Figure 12:**
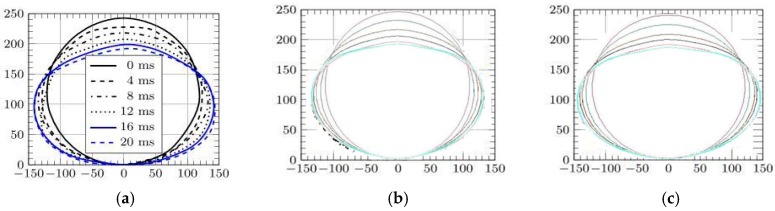
Deformation of the cylinder falling from 1500 mm; (**a**) experiments [42], (**b**) without air, (**c**) with air.

In Figure 11 and Figure 12 this comparison between profiles is shown in detail, considering several times and with both cases of cylinders falling from 500 and 1500 mm. In particular, they exhibit the deformed profile of the cylinder during an observation period of 20 ms, increasing the observation time for 4 ms. Images show a similar trend between experiments and simulations with a better correspondence in the case of models where the air effect is considered.

**Figure 13:**
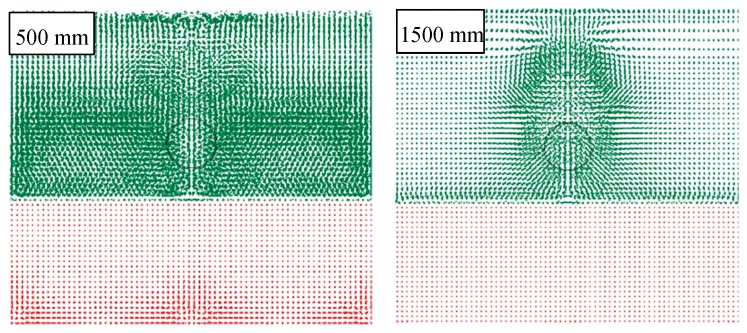
Effect of different falling heights on the air particles (500 and 1500 mm).

It can be also noted that, as the drop height and subsequent velocity of impact increases, the influence of air on deformation decreases because the inertial force far outstrips the contact forces between air SPH particles and shell. This phenomenon also emerged while observing the track of the object through the air, as reported in Figure 13, where simulations are compared considering two different falling heights. In particular, an object that goes through the atmosphere at a greater speed has a more limited influence in terms of distance of the particles affected. This effect is correctly foreseen by the FEM-SPH model, which also foresees the onset of the first effects in the underlying fluid (water) caused by the pressure of the moving object in the overlying fluid (air). It is in fact possible to observe the first movements of water particles both on the vertical of the object and in the corners of the box, thus unloading the overpressure on the edge.

### 4.3. Algorithm Efficacy

A final aspect which has to be considered while using these coupled models deals with the computational time, ras eported in Table 3 (referred to the performance offered by an Intel i5-4590 3.3 GHz processor with 32 GB). As it can be highlighted, changes in the physical aspects of the simulation, such as differences in the impact heights/speeds have no practical influence on the computation. Changes in the SPH parameters can significantly affect the algorithms (as in the case of smoothing lengths, where +20% increases the computation time up to +79%). But, a tangible difference is represented by the inclusion of air particles in the model, a situation that significantly increases the computational time up to 2.5 times.

## 5. Conclusions

This paper presented an analysis on the influence of air in the fluid–structure interaction (FSI) by coupling FEM and SPH. The advantage of using the Lagrangian material framework in FSI is that it offers insight into loads that arise from contact with highly-deformable solids or fluids. For instance, this methodology is used in the study of bird strikes, projectile impacts, explosions, and crashes on water or other surfaces. Drop tests are experiments used to assess airframe crashworthiness, and material and joints behavior under such extreme loads. As in other branches of industry, numerical simulations can be used to reduce cost and time for novel design developments, and the coupling of FEM with the mesh-free method allows for a realistic prediction of loads. Experimental measures and numerical predictions are displayed side by side, permitting their direct comparison, but also highlighting aspects worthy of attention regarding the two numerical models based on the SPH-FEM coupling used here: one with air SPH pseudo-particles and the other without them. In this paper, a significant correspondence between experimental and numerical results was shown. The down side of the SPH mesh-free method is the need for frequent search for neighboring particles and small time-step, resulting in enormous computational requirements and long analysis time. This leads many researchers and engineers to neglect air in their model in order to obtain a model with as few SPH particles as possible. As this investigation shows, air is not necessary for accurate stress prediction, but it is required if the behavior of the structure after the initial water impact is studied. An exact model with additional air SPH particles sinks slowly in comparison to an original model where air bubbles stick to the outer surface of the cylinder and air also prevents the flooding of water into fuselage interior. FEM-SPH can also predict the occurrence of specific phenomena like cavitation. Yet to have more authentic modeling of such problems, further advances in SPH method are required, such as the implementation of phase change from water to vapor within the constitutive model of the fluid.

## Figures and Tables

**Table 1:** Selected contact parameters as defined in LS-DYNA for FEM.

*CONTACT_AUTOMATIC_NODES_TO_SURFACE					
FS	Static Coefficient of Friction	0	SFST	Scale factor for slave surface	1
FD	Dynamic Coefficient of Friction	0	SFMT	Scale factor for master surface	1
DC	Exponential decay coefficient	0	FSF	Coulomb frictional scale factor	1
VC	Coefficient for viscous friction	0	VSF	Viscous frictional Scale factor	1
VDC	Viscous damping Coefficient	0	SOFT	Soft constraint option (Penalty)	0
PENCHK	Small penetration in Contact	0	SOFSCL	Scale factor for constraint forces	0.1
BT	Birth time	0	LCIDAB	Load curve ID defining airbag	0
DT	Death time	10^20^	MAXPAR	Maximum parametric coordinate	1.025
SFS	Scale factor on slave	1–0.5	SBOPT	Segment-based contact options	2
SFM	Scale factor on master	1–0.5	DEPTH	Search depth in automatic contact	2
SST	Optional thickness for slave	-	BSORT	Number of cycle between bucket sorts	-
MST	Optional thickness for master	-	FRCFRQ	Number of cycle between contact force	1

**Table 2:** Selected contact parameters as defined in LS-DYNA for SPH.

*DEFINE_SPH_TO_SPH_COUPLING					
PFACT	Penalty scale factor	0.7	DFACT	Penalty scale factor for contact damping coefficient	1
SRAD	Scale factor nodes to nodes contact	1.0			

**Table 3:** Analysis time for different smoothing length (*h*), drop height and air particles cases (with values expressed by a multiplication factor respect to a base-time (*t_0_*) equal to 116 minutes for an Intel i5-4590 3.3 GHz processor with 32 GB).

	Smoothing Length				Drop Height
Air	1.0	1.1	1.2	Δ	
without	1	1.12	1.43	43%	500 mm
with	2.68	3.22	4.06	51%	
Δ	168%	187%	184%		
without	0.97	1.13	1.50	54%	1500 mm
with	2.76	3.30	4.95	79%	
Δ	183%	192%	230%		
